# Glucagon-Like Peptide-1 Receptor Agonists in Cognitive and Mental Health Disorders: A Comprehensive Review of Pre-Clinical and Clinical Evidence

**DOI:** 10.1192/bjo.2025.10793

**Published:** 2025-06-20

**Authors:** Ana Ghenciulescu, Riccardo De Giorgi, Oliwia Dziwisz, Catherine Harmer

**Affiliations:** 1 Department of Psychiatry, University of Oxford, Oxford, United Kingdom; 2 Oxford Health NHS Foundation Trust, Oxford, United Kingdom

## Abstract

**Aims:** Glucagon-like peptide-1 receptor agonists (GLP-1RAs) such as semaglutide are considered breakthrough drugs in the management of diabetes and obesity. Beyond their metabolic benefits, these pharmacological agents interact with biological pathways that may influence brain function, and are therefore increasingly being investigated for possible repurposing in psychiatric and neurological disorders. This review aims to synthesise pre-clinical and clinical evidence on the effects of GLP-1RAs across a range of cognitive and mental health conditions, comprehensively assessing their therapeutic potential and translational implications for psychiatric practice.

**Methods:** A systematic literature search was conducted across multiple databases, using a broad search algorithm to maximise the scope of the evidence synthesis. Two researchers independently screened titles and abstracts, assessed full texts for eligibility, and extracted relevant data. Results were considered in a narrative and visual synthesis based on emerging categories: studies were divided into mechanistic and clinical evidence, and organised based on broad diagnostic domains (cognitive disorders, substance use disorders, psychotic disorders, mood and anxiety disorders, and eating disorders). Clinical evidence, including meta-analyses, randomised controlled trials (RCTs), and observational studies, was critically appraised and ranked by hierarchy of evidence.

**Results:** The main themes emerging from the 280 pre-clinical and 96 clinical studies identified consist of the potential benefits of GLP-1RAs in neurocognitive disorders (reducing dementia risk and cognitive impairment in various cohorts) and in substance use disorders. Mechanistic evidence suggests these are mediated through their multimodal neuroprotective effects (including via anti-inflammatory pathways) and by dopaminergic modulation of reward mechanisms, respectively. In psychotic disorders, GLP-1RAs primarily mitigate antipsychotic-induced metabolic side effects, with minimal evidence for direct effects on psychosis itself. Findings in mood and anxiety disorders are inconclusive, with some studies reporting antidepressant properties while others show no clear benefit. Evidence in eating disorders is scarce, but suggests GLP-1RAs may influence binge-eating behaviour, aligning with pre-clinical findings on their influence on appetite and reward regulation.

**Conclusion:** Extensive pre-clinical literature on GLP-1RAs provides strong mechanistic support for their putative benefits in Psychiatry, particularly in cognitive and reward-related disorders. However, clinical studies have yet to fully confirm these effects, highlighting the need for high-quality, targeted trials to distinguish direct mental health effects from secondary metabolic improvements. As enthusiasm about the promise of GLP-1RAs continues to grow in both the scientific community and in the media, it is crucial to approach their adoption in Psychiatry cautiously, and focus on robust translational research to establish their long-term efficacy and safety in patients with mental health conditions.

